# Lipopolysaccharide released from gut activates pyroptosis of macrophages via Caspase 11‐Gasdermin D pathway in systemic lupus erythematosus

**DOI:** 10.1002/mco2.610

**Published:** 2024-06-14

**Authors:** Yue Xin, Changxing Gao, Lai Wang, Qianmei Liu, Qianjin Lu

**Affiliations:** ^1^ Hospital for Skin Diseases Institute of Dermatology Chinese Academy of Medical Sciences and Peking Union Medical College Nanjing China; ^2^ Key Laboratory of Basic and Translational Research on Immune‐Mediated Skin Diseases Chinese Academy of Medical Sciences Nanjing China; ^3^ Jiangsu Key Laboratory of Molecular Biology for Skin Diseases and STIs Chinese Academy of Medical Sciences Nanjing China; ^4^ Hunan Key Laboratory of Medical Epigenomics, The Second Xiangya Hospital, Central South University, Changsha, China Changsha China

**Keywords:** Caspase 11, Gasdermin D, lipopolysaccharide, pyroptosis, systemic lupus erythematosus, wedelolactone

## Abstract

Noncanonical pyroptosis is triggered by Caspase 4/5/11, which cleaves Gasdermin D (GSDMD), leading to cell lysis. While GSDMD has been studied previously in systemic lupus erythematosus (SLE), the role of pyroptosis in SLE pathogenesis remains unclear and contentious, with limited understanding of Caspase 11‐mediated pyroptosis in this condition. In this study, we explored the level of Caspase 11‐mediated pyroptosis in SLE, identifying both the upstream pathways and the interaction between pyroptosis and adaptive immune responses. We observed increased Caspase 5/11 and GSDMD‐dependent pyroptosis in the macrophages/monocytes of both lupus patients and mice. We identified serum lipopolysaccharide (LPS), released from the gut due to a compromised gut barrier, as the signal that triggers Caspase 11 activation in MRL/lpr mice. We further discovered that pyroptotic macrophages promote the differentiation of mature B cells independently of T cells. Additionally, inhibiting Caspase 11 and preventing LPS leakage proved effective in improving lupus symptoms in MRL/lpr mice. These findings suggest that elevated serum LPS, resulting from a damaged gut barrier, induces Caspase 11/GSDMD‐mediated pyroptosis, which in turn promotes B cell differentiation and enhances autoimmune responses in SLE. Thus, targeting Caspase 11 could be a viable therapeutic strategy for SLE.

## Graphical abstract

We found that the LPS translocated from the gut was able to trigger Caspase 11/GSDMD induced pyroptosis of macrophages in lupus‐prone mice. The pyroptotic macrophages were able to facilitate the differentiation of plasma cell, by which process the intestinal disorders contributed to the development of lupus. We also confirmed that Caspase 11‐inhibition was a potential therapy for lupus.

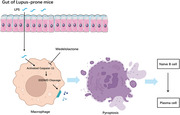

## 1 INTRODUCTION

Systemic lupus erythematosus (SLE) is an autoimmune disease characterized by overactivity of the immune system and the presence of autoantibodies and resultant multisystemic damages, which is of great heterogeneity in clinical manifestations.^1^ The etiology of SLE is intricate, involving genetic predispositions and environmental factors.^2^, ^3^, ^4^ One of the hypotheses proposes that the surplus cell debris produced by increased cell death initiates immune overactivity and induces loss of immunological tolerance.^5^ Apoptosis was thought as the primary death form in SLE pathogenesis, during which the nuclear antigens are released and accessible to the immunocytes due to clearance deficiency.^6^, ^7^ Recently, neutrophil ferroptosis has been found as an important driver in SLE onset, contributing to the presence of lupus manifestations.^8^ However, the involvement of another programmed cell death, pyroptosis, has not been clarified in SLE.

Pyroptosis is a type of programmed cell death distinguished by the gasdermin protein family's perforation and disruption of the cell membrane. In the canonical pathway, inflammasome sensors are activated by damage‐associated molecular patterns (DAMPs) and pathogen‐associated molecular patterns, recruiting apoptosis‐associated speck‐like protein containing a Caspase‐activation and recruitment domain (ASC) and pro‐Caspase 1 to form the inflammasome complex. Upon activation, Caspase 1 cleaves Gasdermin D (GSDMD), producing the N‐terminal fragment of GSDMD, which assembles into pores on the plasma membrane.^9^ In the noncanonical pathway, lipopolysaccharide (LPS) directly triggers Caspases 4/5 (in humans) and Caspases 11 (in mice), leading to GSDMD cleavage and subsequent cell disruption.^10^ Caspase 4/5 and the murine analogue Caspase 11 are specific receptors of cytoplasmic LPS. Except for the Caspase family, other upstream inflammatory signals including cathepsin G and neutrophil elastase in azurophil granules and mitochondrial reactive oxygen species (mtROS) can also promote GSDMD oligomerization.^11^ Along with the pore formation, the preinflammatory cytokines mainly including IL‐1β and IL‐18, alarmins, and nucleic acids are released as DAMPs to further promote inflammation.^12^, ^13^ Previous studies have found some links between the inflammatory process of pyroptosis and SLE development.^14^, ^15^ There are signs of elevated GSDMD‐mediating pyroptosis exists both in patients and mice with lupus. Nevertheless, *GSDMD* ^−/−^ mice exhibited increased mortality, exacerbated nephritis, and enhanced production of autoantibodies when exposed to imiquimod.^14^ At present, the role of GSDMD and pyroptosis is controversial in SLE development and the related upstream pathways have not been confirmed.

In this study, we confirmed the enhanced Caspase 5/11‐induced GSDMD activation in macrophages of lupus patients and lupus‐prone MRL/lpr mice. Next, we identified the serum LPS released from the gut due to the damaged gut barrier as upstream signals triggering Caspase 11‐induced pyroptosis. Importantly, we investigated the effects that pyroptotic macrophages exerted on the differentiation of T and B lymphocytes. Moreover, we evaluated the therapeutic potential of Caspase 11 inhibition as well as the gut barrier repair by the application of antibiotics in MRL/lpr mice.

## 2 RESULTS

### 2.1 Caspase 5/11‐mediated pyroptosis is elevated in monocytes/macrophages of SLE patients and MRL/lpr mice

**FIGURE 1:**
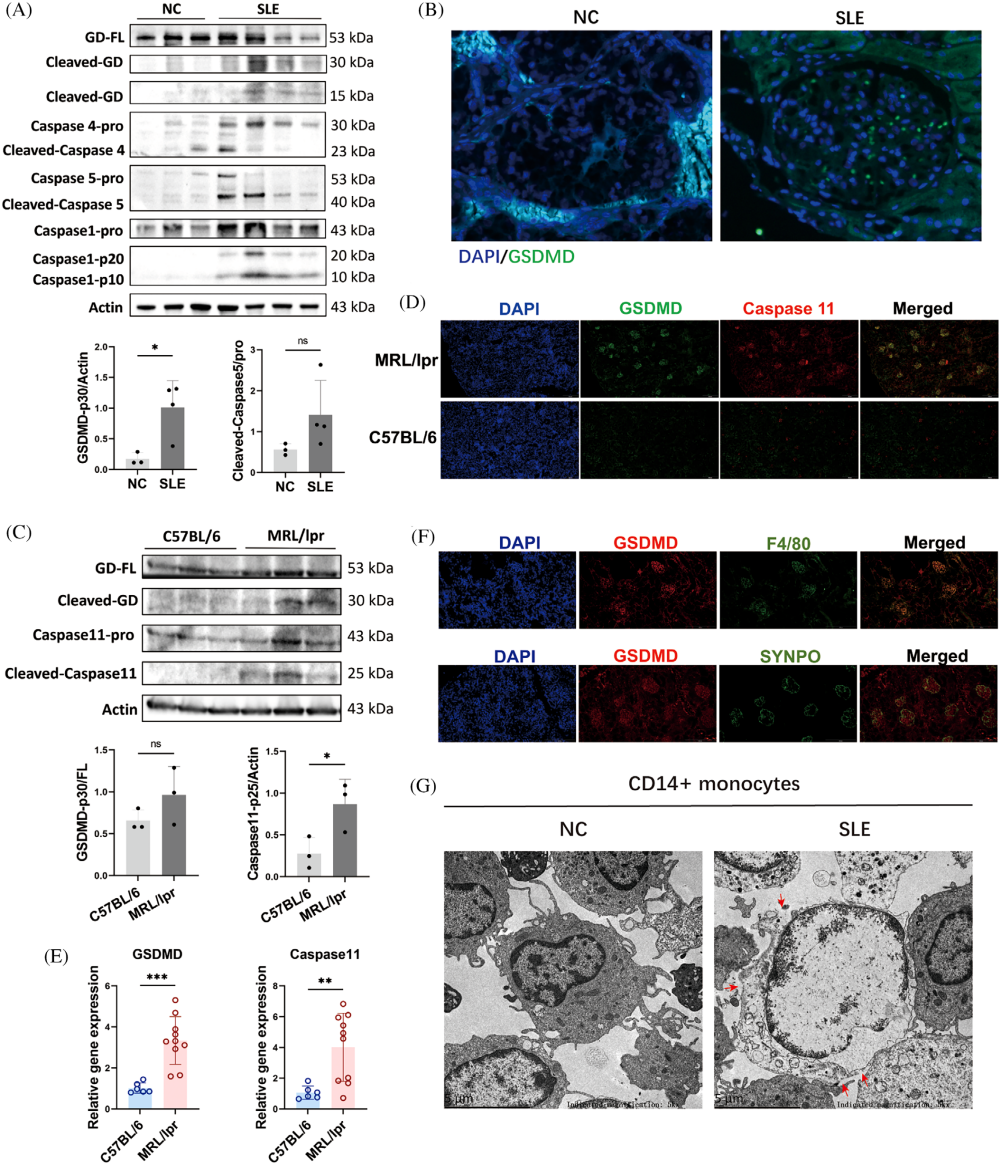
Caspase 5/11‐mediated pyroptosis is elevated in monocytes/macrophages of systemic lupus erythematosus patients and lupus mice. (A) Western blot and semi‐quantified analysis of Caspase 1, Caspase 4, Caspase 5, Gasdermin D (GSDMD), and their cleavage forms in peripheral blood mononuclear cells (PBMCs) isolated from systemic lupus erythematosus (SLE) patients (*n* = 4) and normal controls (NC, *n* = 3). (B) Immunohistochemistry of GSDMD in renal biopsy samples from lupus nephritis patients and the renal peritumoral tissue from renal tumor patients as the control. (C) Western blot and semi‐quantified analysis of Caspase 11, GSDMD, and their cleavage forms in kidneys of MRL/lpr mice (*n* = 3) and C57BL/6 mice (*n* = 3). (D) Immunofluorescence of GSDMD and Caspase 11 in kidneys of MRL/lpr mice. Scale bar: 200 μm. (E) Caspase 11 and GSDMD mRNA expression in kidneys of MRL/lpr mice (*n* = 10) and C57BL/6 mice (*n* = 6). (F) Immunofluorescence colocalization of GSDMD and F4/80 or synaptopodin (SYNPO) in kidneys of MRL/lpr mice. Scale bar: 200 μm. (G) Representative transmission electron micrographs of CD14^+^ monocytes isolated from PBMCs of SLE patients and normal controls. Red arrowhead: membrane pores. Scale bar: 5 μm. Data are presented as mean ± SD. Significance was examined with an unpaired two‐sided Student's *t*‐test. *p < 0.05, **p < 0.01, ***p < 0.001; ns, not significant.

To demonstrate that accelerated pyroptosis occurs in individuals with SLE, we initially examined the cleavage of GSDMD, a definitive marker of pyroptosis, in the peripheral blood mononuclear cells (PBMCs) of SLE patients. We enrolled three healthy individuals and four patients with varying Systemic Lupus Erythematosus Disease Activity Index (SLEDAI) to assess GSDMD cleavage in PBMCs. Notably, increased cleavages of Caspase 5 and GSDMD were observed in the PBMCs of SLE patients compared with healthy controls (Figure 1A). However, a positive correlation between GSDMD‐mediated pyroptosis and SLEDAI in SLE patients was not established. Multiplex immunohistochemistry (mIHC) revealed that renal biopsy samples from lupus nephritis (LN) patients showed heightened expression of GSDMD in the glomeruli, using renal peritumoral tissue from renal tumor patients as controls (Figure 1B). The Caspase 4 and Caspase 5 expressions were also significantly increased in LN (Figure S1). At the protein level, there was an increase in the cleavages of Caspase 11 and GSDMD in the kidneys of lupus mice compared with healthy control (C57BL/6 mice) (Figure 1C). Similarly, elevated expression and colocalization of GSDMD and Caspase 11 were detected in kidneys from MRL/lpr mice (Figure 1D,E). Furthermore, we aimed to identify which cell types predominantly undergo pyroptosis in SLE. Contrary to previous reports,^16^ our findings from immunofluorescence colocalization indicated that in the glomeruli of MRL/lpr mice, macrophages marked by F4/80, rather than podocytes marked by synaptopodin (SYNPO), were undergoing pyroptosis (Figure 1F). The CD14^+^ cells in PBMCs are circulating monocytes, which are the precursors of macrophages in tissues. Transmission electron microscopy (TEM) demonstrated multiple pores in the membranes of isolated CD14^+^ monocytes from the PBMCs of SLE patients, consistent with the morphological characteristics of pyroptosis^17^, ^18^ (Figure 1G). These results collectively suggest that Caspase 5/11 and GSDMD‐mediated pyroptosis is significantly elevated in monocytes/macrophages of lupus mice and SLE patients.

### 2.2 Pyroptosis of J774A.1 and RAW264.7 cells is induced by incubation with serum from MRL/lpr mice

**FIGURE 2:**
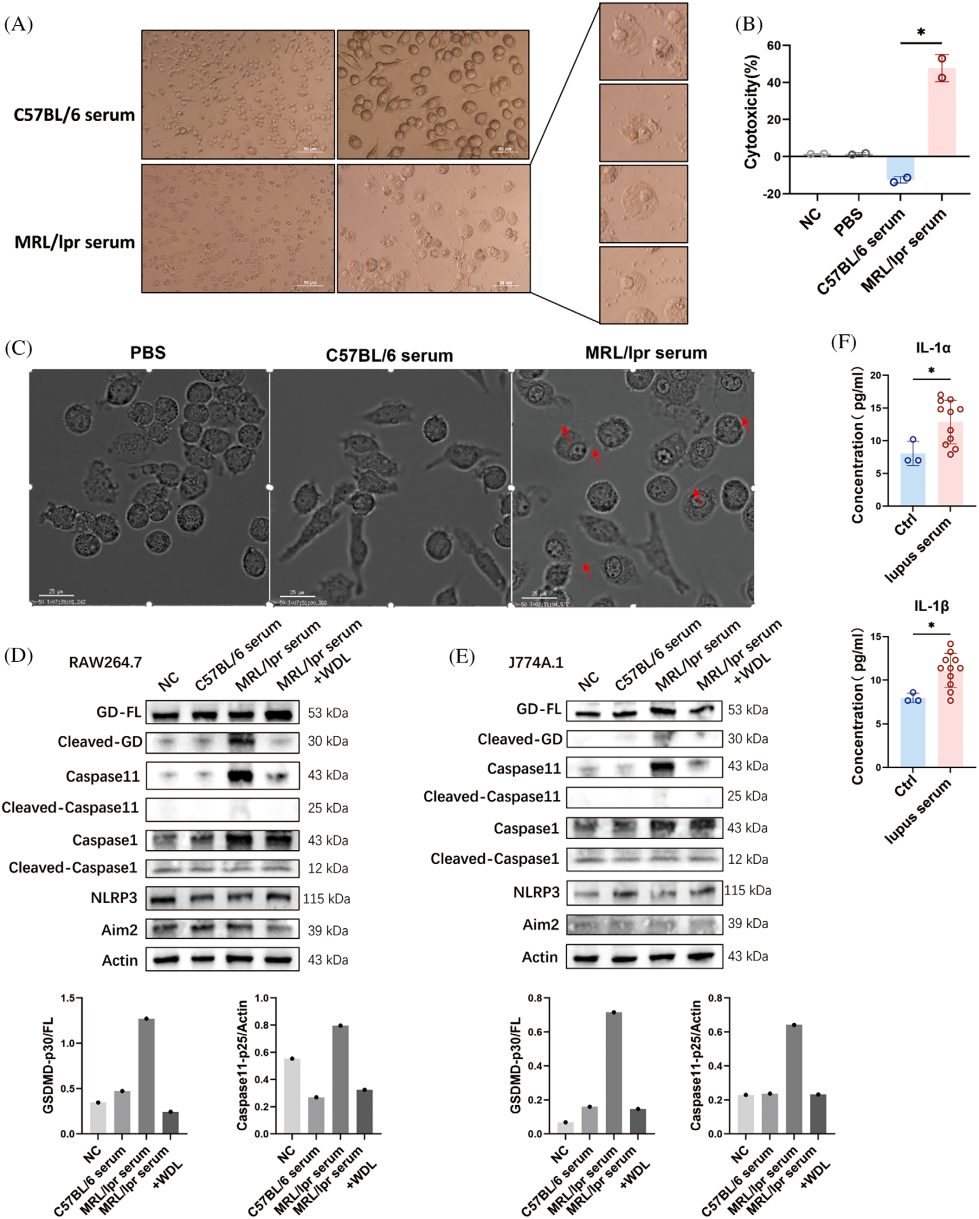
Pyroptosis of J774A.1 and RAW264.7 cells is induced by incubation of serum from MRL/lpr mice. (A) Representative images by light microscopy of J774A.1 cells that were cultured by 20% serum from C57BL/6 or MRL/lpr mice for 24 h. Scale bar: 50 μm, 25 μm. (B) Lactate dehydrogenase (LDH) releasing assay of J774A.1 cells that was cultured by 20% PBS, serum of MRL/lpr mice, and serum of C57BL/6 mice for 24 h. The calculation of percentages of cytotoxicity was analyzed on maximum LDH release from cells treated with 1% Triton X‐100. (C) Time‐lapse microscopy assay of J774A.1 cells that were cultured by 20% PBS, MRL/lpr mice serum, and C57BL/6 mice serum for 24 h. Red arrowhead: membrane blebbing. Scale bar: 25 μm. (D) Western blot and semi‐quantified analysis of Nlrp3, Aim2, Caspase1, Caspase 11, Gasdermin D (GSDMD), and their cleavage forms in RAW264.7 cells that were cultured by 20% PBS (NC), C57BL/6 mice serum, MRL/lpr mice serum, and MRL/lpr mice serum + wedelolactone (WDL) for 24 h. (E) Western blot and semi‐quantified analysis of Nucleotide‐binding oligomerization domian like receptor family pyrin domian containing (Nlrp3), Absent in melanoma 2 (Aim2) , Caspase1, Caspase 11, GSDMD, and their cleavage forms in J774A.1 cells that were cultured by 20% PBS (NC), C57BL/6 mice serum, MRL/lpr mice serum, and MRL/lpr mice serum + WDL for 24 h. (F) Supernatant IL‐1α and IL‐1β levels after J774A.1 and RAW264.7 cells cultured by 20% PBS (Ctrl, *n* = 3) and 20% MRL/lpr mice serum (*n* = 11) for 24 h. Data are presented as mean ± SD. Significance was examined with an unpaired two‐sided Student's *t*‐test. *p < 0.05; ns, not significant.

We hypothesized that the serum from lupus mice could induce pyroptosis in macrophages. To test this hypothesis, we analyzed morphological changes, cytotoxic reactions, and protein expression in J774A.1 cells cultured with serum [at a concentration of 20% (v/v)] from MRL/lpr mice. Morphological observations using light microscopy revealed that J774A.1 cells exhibited a pyroptosis‐like phenotype, characterized by swelling, rounding, and the release of cell contents, which were not observed in cells cultured with serum from C57BL/6 mice (Figure 2A). The induction of pyroptosis was further supported by a lactate dehydrogenase (LDH) release assay, a widely accepted method for quantitatively assessing cell membrane integrity and viability. Incubation with lupus serum significantly increased LDH release from J774A.1 cells, indicating a stronger capacity to induce cell lysis compared with serum from healthy mice (Figure 2B). The dynamic processes were documented using a time‐lapse microscopy assay, with videos provided in the Supporting Information. After 4 h of incubation with 20% lupus serum, J774A.1 cells began to exhibit membrane blebbing followed by cell lysis, reaching a cell death rate of 50% by the 7th hour, which was not observed in the C57BL/6 serum and PBS groups (Figure 2C). Notably, elevated cleavages of Caspase 11 and GSDMD, markers of noncanonical pyroptosis, were detected in both J774A.1 and RAW264.7 cells after incubation with 20% lupus serum (Figure 2D,E). Additionally, the levels of supernatant IL‐1α and IL‐1β, specific products of Caspase 11 activation,^19^ were also increased following incubation with lupus serum (Figure 2F). Moreover, the cleavage of GSDMD and Caspase 11 induced by lupus serum was attenuated by the Caspase 11 inhibitor wedelolactone (WDL), a natural compound that suppresses Caspase 11 expression by inhibiting NF‐κB‐mediated transcription,^20^ indicating that the pyroptosis‐inducing effect of lupus serum on macrophages was Caspase 11‐dependent. These findings further confirm the occurrence of Caspase 11‐dependent pyroptosis in the context of lupus and the presence of pyroptosis‐inducing substances in the serum of lupus mice.

### 2.3 Increased serum LPS caused by damaged gut barrier induces pyroptosis of macrophage via TLR 4 and Caspase 11 in MRL/lpr mice

**FIGURE 3:**
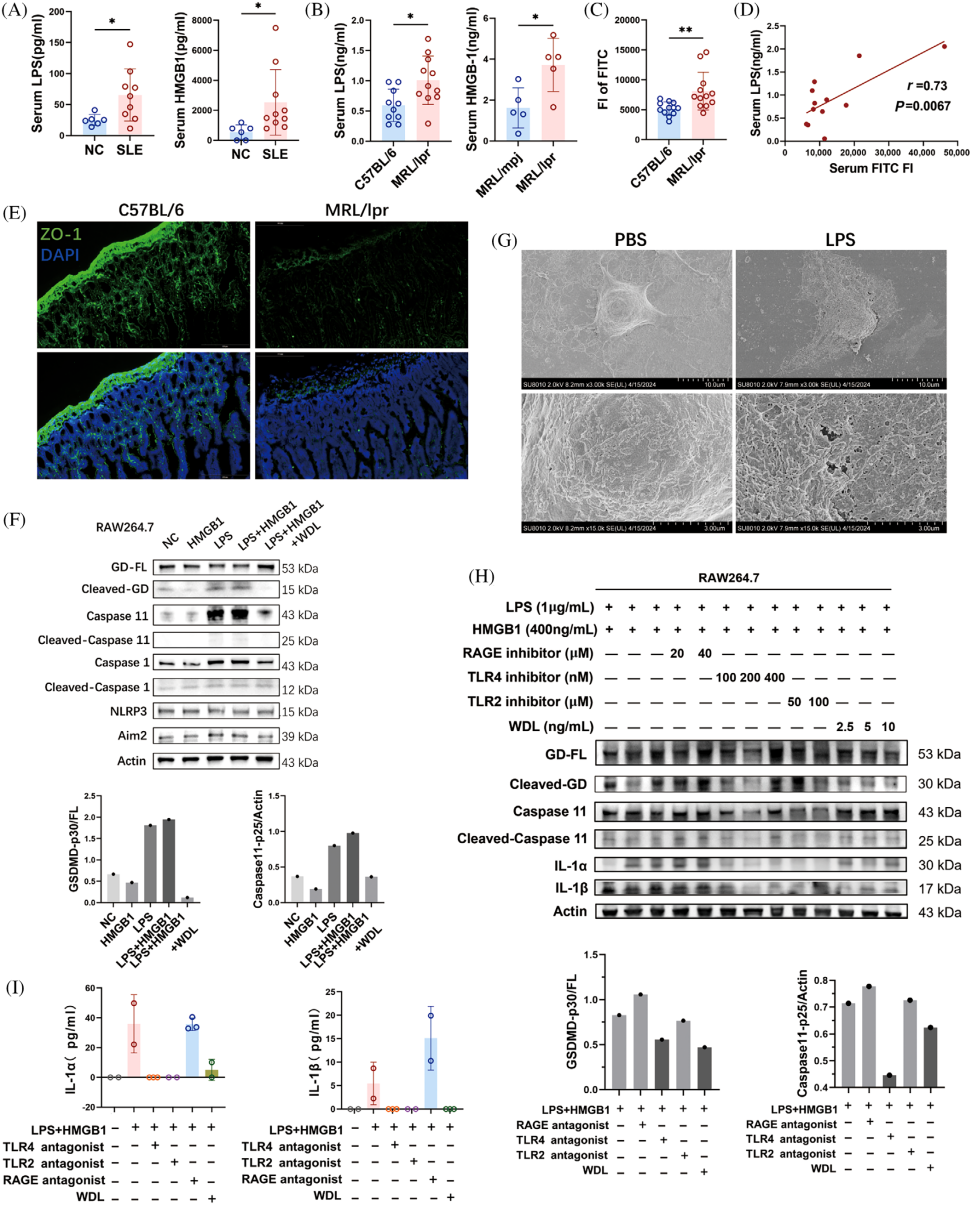
Increased serum lipopolysaccharide caused by damaged gut barrier induces pyroptosis of macrophage in MRL/lpr mice. (A) Serum lipopolysaccharide (LPS) assay of systemic lupus erythematosus (SLE) patients (*n* = 6) and normal controls (NC, *n* = 8); Serum high mobility group box‐1 protein (HMGB1) assay of SLE patients (*n* = 6) and normal controls (NC, *n* = 10). (B) Serum LPS assay of MRL/lpr mice (*n* = 10) and C57BL/6 mice (*n* = 11); serum HMGB1 assay of MRL/lpr mice (*n* = 5) and C57BL/6 mice (*n* = 5). (C) Fluorescence intensity (FI) of serum FITC in C57BL/6 mice and MRL/lpr mice (*n* = 12). (D) Correlation analysis between serum LPS and FI of serum FITC in MRL/lpr mice. (E) Immunofluorescence of Zonula occludens protein‐1 (ZO‐1) in small intestines of C57BL/6 mice and MRL/lpr mice. Scale bar: 200 μm. (F) Western blot and semi‐quantified analysis of Nucleotide‐binding oligomerization domian like receptor family pyrin domian containing (Nlrp3) , Absent in melanoma 2 (Aim2), Caspase1, Caspase 11, Gasdermin D (GSDMD), and their cleavage forms in RAW264.7 cells treated with equivalent PBS (NC), HMGB1 (400 ng/mL), LPS (1 μg/mL), LPS (1 μg/mL) + HMGB1 (400 ng/mL), and LPS (1 μg/mL) + HMGB1 (400 ng/mL) + wedelolactone (WDL, 5 μg/mL) for 24 h. (G) Representative scanning electron micrographs of RAW264.7 cells treated with LPS (1 μg/mL) and equivalent PBS as control. Scale bar: 10 μm, 3 μm. (H) Western blot and semi‐quantified analysis of IL‐1α, IL‐1β, Caspase 11, GSDMD, and their cleavage forms after RAW264.7 cells treated with LPS + HMGB1, LPS + HMGB1 + Toll‐like receptor (TLR) 2 antagonist, LPS + HMGB1 + TLR4 antagonist, LPS + HMGB1 + RAGE antagonist, and LPS + HMGB1 + WDL for 24 h. (I) Supernatant IL‐1α and IL‐1β concentrations after RAW264.7 cells treated with LPS + HMGB1, LPS + HMGB1 + TLR2 antagonist, LPS + HMGB1 + TLR4 antagonist, LPS + HMGB1 + advanced glycation end‐products (RAGE) antagonist, and LPS + HMGB1 + WDL for 24 h. Data are presented as mean ± SD. Significance was examined with an unpaired two‐sided Student's *t*‐test. *p < 0.05, **p < 0.01.

We further explored the agents inducing pyroptosis in lupus mice. Caspase 4, 5, and their murine counterpart Caspase 11, serve as specific receptors for cytoplasmic LPS. Previous study demonstrated that LPS directly interacts with Caspase 4/5/11 under the mediation of the Caspase‐activation and recruitment domain of caspases, leading to their oligomerization and activation.^21^ They also found that the induction of pyroptosis by cytoplasmic LPS was prevented in Caspase 11 knockout macrophages and restored upon stable re‐expression of Caspase 11.^21^ Additionally, Deng et al.^22^ found that High mobility group box‐1 protein (HMGB1) was essential for LPS internalization and Caspase11‐dependent pyroptosis. HMGB1 facilitated the internalization of LPS via the receptor for advanced glycation end‐products (RAGE) in macrophages. We detected an elevated LPS and HMGB1 level in the serum of SLE patients (Figure 3A) and MRL/lpr mice (Figure 3B). We hypothesized that the increased serum LPS originated from the intestines due to compromised gut barrier integrity in lupus mice. To test intestinal permeability, fluorescein isothiocyanate (FITC)‐conjugated dextran was administered into mice by gavage, and serum FITC intensity was measured.^23^ The FITC–dextran penetration test indicated that MRL/lpr mice exhibited increased gut permeability compared with C57BL/6 mice (Figure 3C). The mean fluorescence intensity of serum dextran, indicative of gut leakage, positively correlated with serum LPS levels (Figure 3D), supporting our hypothesis that the serum LPS was released from the gut. Additionally, in MRL/lpr mice, the levels of Zonula Occludens protein 1 (ZO‐1), an important intestinal tight junction protein, was found to be lower than those in age‐matched C57BL/6 mice (Figure 3E). This decrease in ZO‐1 expression aligns with the observed loss of intestinal barrier integrity in the MRL/lpr mice.

To verify whether LPS and HMGB1 could induce Caspase 11‐dependent pyroptosis in macrophages. we treated RAW264.7 cells with LPS and HMGB1, both individually and in combination, and assessed the protein levels of key markers of noncanonical pyroptosis. The results indicated that HMGB1 alone did not alter the cleavage levels of Caspase 11 and GSDMD in macrophages (Figure 3F). Treatments with both LPS alone and LPS combined with HMGB1 resulted in elevated cleavages of Caspase 11 and GSDMD, suggesting that LPS is the primary activator in Caspase 11‐dependent pyroptosis. In addition, Caspase 11 inhibitor WDL effectively reduced the levels of pro‐Caspase 11, the cleavage of Caspase 11, and the cleavage of GSDMD induced by LPS, implying that the LPS‐induced pyroptosis was Caspase 11 dependent (Figure 3F). Scanning electron microscopy was employed to evaluate the detailed surface morphology of RAW264.7 cells following treatment with 1 μg/mL LPS or an equivalent PBS as control (Figure 3G). Unlike the control group, which displayed a flat surface with abundant extracellular vesicles, LPS‐treated cells exhibited multiple pores or pits on their membrane, indicative of structural compromise. Due to the varying sizes of these pores, the cell surface appeared rough, and cell volume increased following LPS treatment. This morphological change supported that LPS induced pyroptosis in RAW264.7 cells.

We further investigated potential mediating receptors in LPS‐induced pyroptosis using receptor antagonists. In experiments with RAW264.7 cells treated with LPS and HMGB1, antagonists for Toll‐like receptor (TLR) 2, TLR4, and RAGE were introduced. The results revealed that the TLR4 antagonist at a concentration of 200 nM effectively inhibited the cleavage of Caspase 11 and GSDMD (Figure 3H), along with the secretion of IL‐1α and IL‐1β induced by LPS (Figure 3H,I). This indicates that TLR4 mediates LPS‐induced Caspase 11/GSDMD‐dependent pyroptosis. Additionally, The Caspase 11 inhibitor WDL effectively attenuated the enhanced cleavage of GSDMD, as well as the production of IL‐1α and IL‐1β (Figure 3H,I). These findings collectively demonstrate that the disruption of the intestinal barrier in MRL/lpr mice leads to the migration of LPS originating from the gut into the systemic circulation, subsequently inducing Caspase 11/GSDMD‐dependent pyroptosis in macrophages through TLR4 mediation.

### 2.4 Caspase 1‐dependent canonical pyroptosis pathway involvements in SLE

**FIGURE 4:**
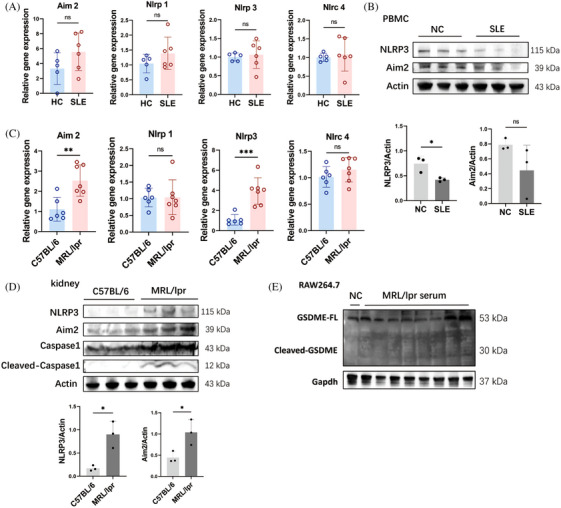
Canonical pyroptosis pathway involvement in systemic lupus erythematosus. (A) The mRNA expression of inflammasomes related with canonical pyroptosis pathway in peripheral blood mononuclear cells (PBMCs) isolated from systemic lupus erythematosus (SLE) patients (*n* = 6) and normal controls (NC, *n* = 5). (B) Western blot and semi‐quantified analysis of Nucleotide‐binding oligomerization domian like receptor family pyrin domian containing 3 (NLRP3) and Absent in melanoma 2 (Aim2) in PBMCs isolated from SLE patients (*n* = 3) and normal controls (NC, *n* = 3). (C) The mRNA expression of inflammasomes related with canonical pyroptosis pathway in kidneys of MRL/lpr mice (*n* = 7) and C57BL/6 mice (*n* = 6). (D) Western blot and semi‐quantified analysis of NLRP3, Aim2, Caspase 1, and cleaved Caspase 1 in kidneys of MRL/lpr mice (*n* = 3) and C57BL/6 mice (*n* = 3). (E) Western blot of Gasdermin E (GSDME) and cleaved‐GSDME in RAW264.7 cells cultured by 20% PBS (NC) and MRL/lpr mice serum for 24 h. Data are presented as mean ± SD. Significance was examined with an unpaired two‐sided Student's *t*‐test. *p < 0.05, **p < 0.01, ***p < 0.001; ns, not significant.

In the canonical pyroptosis pathway, inflammasomes such as Nucleotide‐binding oligomerization domian like receptor family pyrin domian containing 1(Nlrp1), Absent in melanoma 2 (Aim2), Nucleotide‐binding oligomerization domian like receptor family pyrin domian containing 3 (Nlrp3), and NLR family CARD domain‐containing protein 4 (Nlrc4) are activated by various extracellular stimuli to initiate the Caspase 1 cleavage. Initially, we examined the inflammasome expression in PBMCs from SLE patients to investigate the potential role of canonical pyroptosis in SLE. Our findings did not reveal any elevated expressions of Nlrp1, Aim2, Nlrp3, or Nlrc4 in the PBMCs of individuals with SLE compared with the healthy ones (Figure 4A). Similarly, there were no observed elevations of Aim2 and Nlrp3 in protein levels in PBMCs of SLE patients (Figure 4B). However, significant elevated Caspase 1 cleavage was detected in PBMCs of SLE patients (Figure 1A), suggesting an enhancement in Caspase 1‐mediated pyroptosis, which was independent of Nlrp1, Aim2, Nlrp3, and Nlrc4 inflammasomes.

In the kidneys of MRL/lpr mice, both the expressions and protein levels of Aim2 and Nlrp3 were significantly increased (Figure 4C,D). Correspondingly, the cleavage of Caspase 1 was also enhanced in the kidneys of MRL/lpr mice (Figure 4D), indicating an increase in Caspase 1‐dependent pyroptosis in this model. However, incubation with 20% serum from MRL/lpr mice did not enhance the levels of Aim2 and Nlrp3 or the cleavage of Caspase 1 in RAW264.7 and J774A.1 cells (Figure 2D,E). Consistently, neither treatment with LPS alone nor the combination of LPS and HMGB1 increased the levels of Aim2 and Nlrp3, nor the cleavage of Caspase 1 in vitro (Figure 3F). These findings indicate that the elevated GSDMD cleavage induced by lupus serum or LPS was mediated by Caspase 11 activation rather than Caspase 1. Additionally, the cleavage of Gasdermin E (GSDME) was not detected in RAW264.7 cells incubated with lupus serum (Figure 4E).

### 2.5 Pyroptosis of macrophage facilitates the differentiation of B cells

**FIGURE 5:**
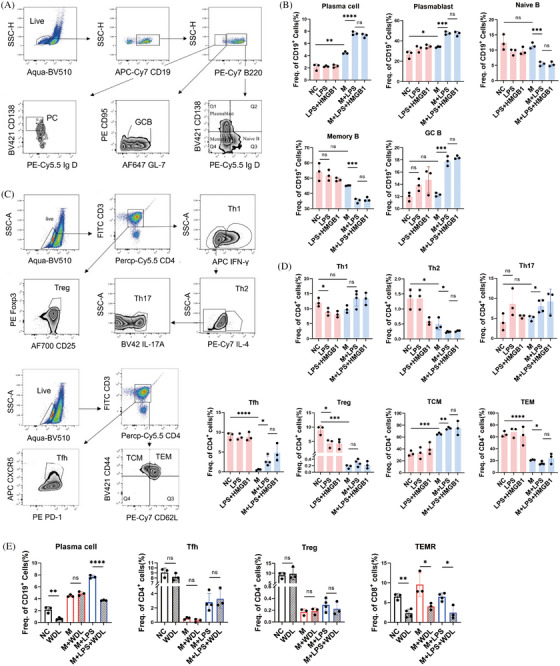
The effects of pyroptotic macrophages on the differentiation of B and T lymphocytes. (A) Flow cytometry plots and gating for plasma cell (PC), plasma blast, naïve B cell, memory B cell, and germinal center B (GCB) cell subsets. (B) The proportions of plasma cell, plasmablast, naïve B cell, memory B cell, and GCB cell subsets in the entire CD19^+^ B cells after activated by R848 and IL‐4 for 48 h. The treatments included blank control (NC), lipopolysaccharide (LPS), high mobility group box‐1protein (HMGB1), LPS+HMGB1, cocultured with autologous peritoneal macrophages (M), peritoneal macrophages + LPS, peritoneal macrophages + LPS + HMGB1 (*n* = 3). (C) Flow cytometry plots and gating for type 1 helper T cell (Th1), type 2 helper T cell (Th2), type 17 helper T cell (Th17), follicular helper T cell (Tfh), regulatory T cell (Treg), central memory T cell (TCM), and effector memory T cell (TEM) subsets. (D) The proportion of Th1, Th2, Th17, Tfh, Treg, TCM, and TEM subsets in the entire CD4^+^ T cells after activated by anti‐CD3 and anti‐CD28 antibodies for 72 h. The treatments included blank control (NC), LPS, HMGB1, LPS + HMGB1, peritoneal macrophage (M), peritoneal macrophage + LPS, peritoneal macrophage + LPS + HMGB1 (*n* = 3). (E) The effects of wedelolactone (WDL) on the differentiations of plasma cell, terminally differentiated effector memory T cells (TEMR), Tfh, and Treg in presence or absence of pyroptotic macrophages. The CD19^+^ B cells and CD4^+^ T cells isolated from the spleens of C57BL/6 mice were treated as follows: blank control (NC), WDL, cocultured with peritoneal macrophages (M), peritoneal macrophages + WDL, peritoneal macrophages + LPS, peritoneal macrophages + LPS + WDL (*n* = 3). Data are presented as mean ± SD. Significance was examined with an unpaired two‐sided Student's *t*‐test. *p < 0.05, **p < 0.01, ***p < 0.001, ****p < 0.0001; ns, not significant.

We investigated the effects of pyroptotic macrophages on adaptive immunocytes to explore the link between pyroptosis and SLE pathogenesis. CD19^+^ B cells were isolated from the spleens of C57BL/6 mice and stimulated in vitro with R848 and murine IL‐4, with or without pyroptotic macrophages. Autologous peritoneal macrophages were isolated and treated with LPS to induce pyroptosis. Flow cytometry plots and gating for plasma cell (PC), plasmablast, naïve B cell, memory B cell, and germinal center B (GCB) cell subsets are shown as Figure 5A. Results showed that coculture with autologous pyroptotic peritoneal macrophages facilitated the differentiation of naive B cells into plasma cells significantly (Figure 5B). when pan B cells were cocultured with macrophages alone, there was a minor rise in the proportions of PCs and plasmablasts with no change observed in the naive, memory B cells, and GCB subgroups compared with the control group. The proportion of mature B cell subsets was not significantly increased by the presence of LPS alone. However, the combination of macrophages and LPS resulted in significant increases in PCs, plasmablasts, and GCB proportions, along with decreases in naive and memory B cell proportions. There was no distinction between the LPS group and the HMGB1+LPS group. The impact of pyroptotic peritoneal macrophages on the differentiation of CD4^+^ T lymphocytes appeared ambiguous (Figure 5C,D). LPS treatment alone did not significantly affect T cell differentiation, except for a reduction in the proportion of regulatory T (Treg) cells. Interestingly, coculture with macrophages inhibited the activation of follicular helper T (Tfh) cells, Treg cells, helper T (Th) 2 cells, and the transition from central memory T (TCM) cells to effector memory T (TEM) cells. In the presence of both macrophages and LPS, the pyroptosis of macrophages induced by LPS counteracted the inhibitory effect on Tfh cell differentiation, although the effect was mild.

Additionally, we examined the effects of the Caspase 11 inhibitor WDL on the differentiation of CD19^+^ B cells and CD4^+^ T cells in the presence or absence of pyroptotic macrophages. Treatment with WDL eliminated the enhancing effect of pyroptotic macrophages on plasma cell differentiation (Figure 5E). Surprisingly, WDL also significantly inhibited plasma cell differentiation even in the absence of pyroptotic macrophages, suggesting its immunosuppressive potential, previously unreported. Furthermore, WDL exerted a strong inhibitory effect on the differentiation of the terminally differentiated effector memory T (TEMR) cell subset and a slight inhibitory effect on TEM cell subset differentiation but had no notable impact on Tfh and Treg subsets.

### 2.6 Caspase 11 inhibitor and antibiotics alleviate lupus symptoms in MRL/lpr mice

**FIGURE 6:**
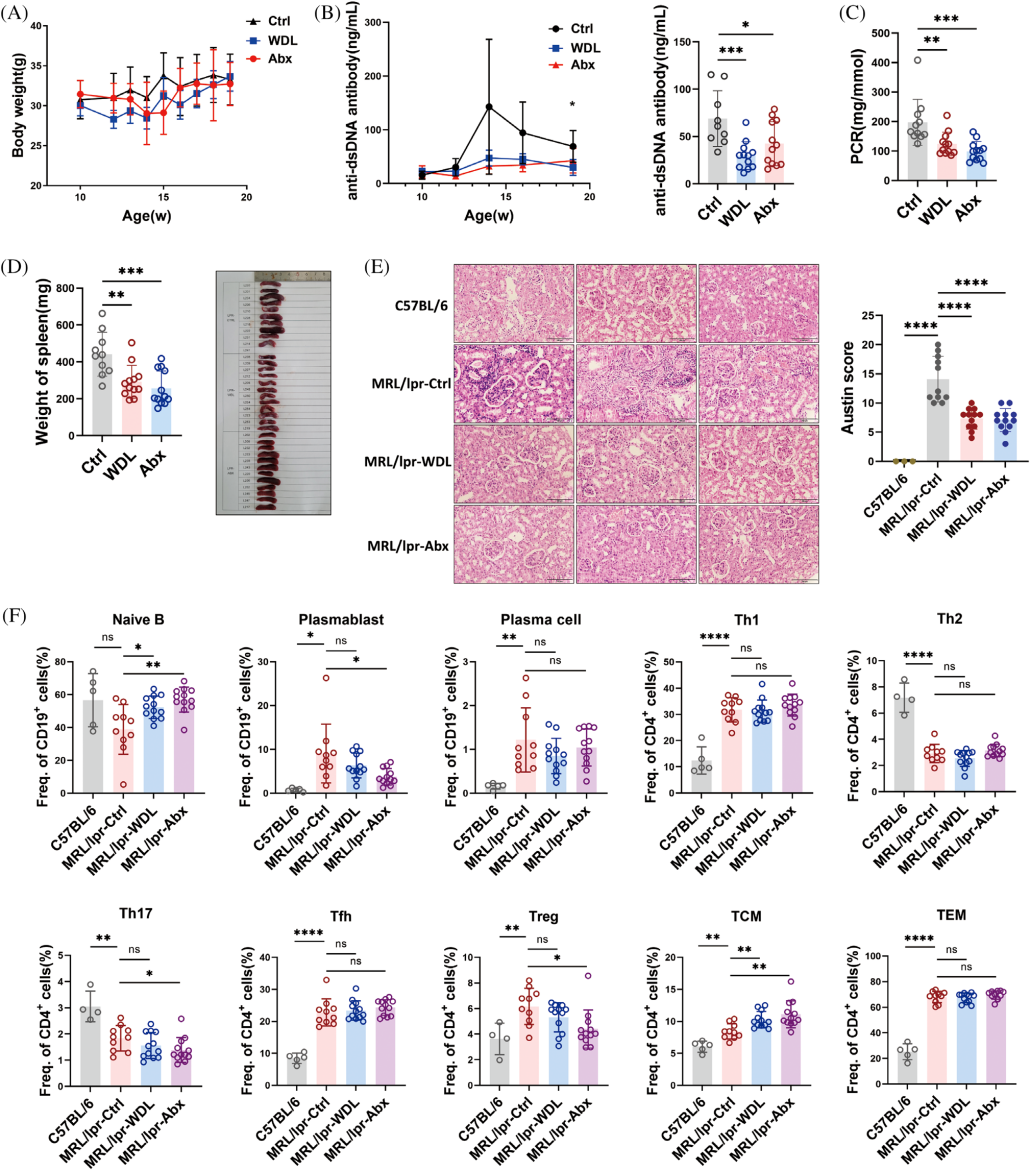
Caspase11 inhibitor and antibiotics alleviate lupus symptoms in MRL/lpr mice. The MRL/lpr mice were treated with a dose of 10 mg/kg wedelolactone (WDL, *n* = 12), combined antibiotics (Abx, *n* = 12), or equivalent vehicle as control (Ctrl, *n* = 11) for 9 weeks. (A) The body weight of mice was measured once a week. (B) (left) The serum anti‐dsDNA antibody titers were examined every 2 weeks. (right) The serum anti‐dsDNA antibody titers of mice at the age of 19 weeks. (C) The protein/creatinine ratio (PCR) of mice at the age of 19 weeks. (D) The morphology and weight of the spleens of mice were recorded immediately after execution. (E) (left) Representative images of renal H&E staining of mice from each group. (right) Austin scores evaluating glomerulonephritis pathology of mice from each group. (F) Proportions of splenic naïve B cell, plasmablast, plasma cell, type 1 helper T cell (Th1), type 2 helper T cell (Th2), type 17 helper T cell (Th17), follicular helper T cell (Tfh), regulatory T cell (Treg), central memory T cell (TCM), and effector memory T cell (TEM) subsets of mice in each group were detected by flow cytometry. Data are presented as mean ± SD. Significance was examined with an unpaired two‐sided Student's *t*‐test. *p < 0.05, **p < 0.01, ***p < 0.001, ****p < 0.0001; ns, not significant.

To determine the role of Caspase 11‐dependent pyroptosis in the progression of SLE, we examined the therapeutic effects of WDL in lupus‐prone mice. MRL/lpr mice were administered 10 mg/kg of WDL or a corresponding vehicle as a control starting at 10 weeks of age and were euthanized 9 weeks later. Additionally, we treated the mice with a combination of antibiotics (ampicillin, metronidazole, neomycin, and vancomycin) via gavage to reduce LPS leakage. Regarding safety, mice in both the WDL‐treated and antibiotics‐treated groups initially lost weight during the early and mid‐stages of treatment, respectively, but subsequently regained it (Figure 6A). Both WDL and antibiotics treatments were effective in suppressing lupus activity, as evidenced by reduced anti‐dsDNA antibody titers (Figure 6B), improved proteinuria levels (Figure 6C), and reduced splenomegaly (Figure 6D). Furthermore, both treatments reduced inflammatory cell infiltration, mesangial cell proliferation, and crescent formation in the kidneys of MRL/lpr mice (Figure 6E). Flow cytometry analysis of lymphocyte subsets in the spleens of these mice revealed that treatment with WDL and antibiotics increased the proportion of naïve B cells and decreased the proportion of plasmablasts, although there were no significant effects on the effector T cell subsets (Figure 6F). Collectively, these findings suggest that inhibition of Caspase 11 and the use of antibiotics can modulate immune function and alleviate lupus symptoms in MRL/lpr mice.

### 2.7 Caspase 11/GSDMD pathway was depressed by WDL and antibiotic treatments in MRL/lpr mice

**FIGURE 7:**
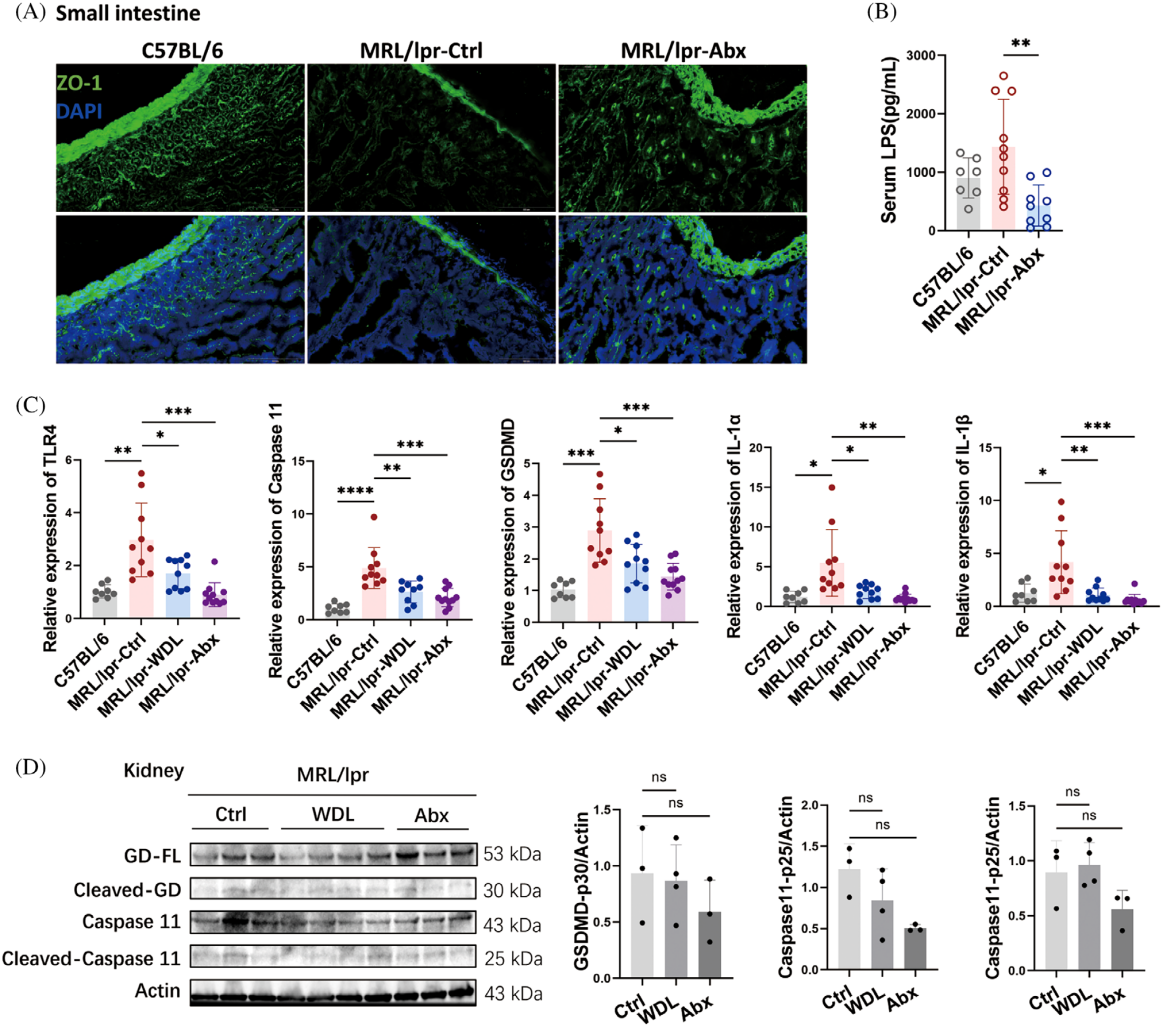
Caspase 11/Gasdermin D pathway was depressed by wedelolactone and antibiotic treatments in MRL/lpr mice. (A) Immunofluorescence of Zonula occludens protein‐1 (ZO‐1) in small intestines of C57BL/6 mice, MRL/lpr mice, and MRL/lpr mice treated by antibiotics (Abx). Scale bar: 200 μm. (B) Serum lipopolysaccharide (LPS) assay of C57BL/6 mice (*n* = 6), MRL/lpr mice (*n* = 10), and MRL/lpr mice treated by antibiotics (Abx, *n* = 9). (C) The mRNA expression of Toll‐like receptor (TLR) 4, Caspase 11, Gasdermin D (GSDMD), IL‐1α, and IL‐1β in kidneys of C57BL/6 mice (*n* = 8), MRL/lpr mice (*n* = 10), MRL/lpr mice treated by wedelolactone (WDL, *n* = 10), and MRL/lpr mice treated by antibiotics (Abx, *n* = 11). (D) Western blot and semi‐quantified analysis of Caspase 11, GSDMD, and their cleavage forms in kidneys of MRL/lpr mice, MRL/lpr mice treated by WDL, and MRL/lpr mice treated by antibiotics (Abx). Data are presented as mean ± SD. Significance was examined with an unpaired two‐sided Student's *t*‐test. *p < 0.05, **p < 0.01, ***p < 0.001, ****p < 0.0001; ns, not significant.

We further explored the mechanisms underlying the effectiveness of WDL and antibiotics in treating SLE, particularly whether these treatments are associated with the inhibition of LPS and Caspase 11 as hypothesized. Immunofluorescence analysis revealed a significant increase in ZO‐1 in the small intestinal epithelium of the antibiotic‐treated group compared with the vehicle control group (Figure 7A), accompanied by a substantial decrease in serum LPS levels (Figure 7B). Both WDL and antibiotics significantly reduced the expression of TLR4, Caspase 11, GSDMD, and the downstream inflammatory cytokines IL‐1α and IL‐1β in the kidneys of the treated mice (Figure 7C). Additionally, the protein levels of Caspase 11, Caspase 11‐p25, and GSDMD‐p30 in the kidneys exhibited a decreasing trend following treatment with WDL and antibiotics (Figure 7D). These findings indicate that WDL and antibiotic treatments alleviate lupus symptoms by reducing Caspase 11‐induced cell pyroptosis, thus confirming our initial speculation regarding their mechanism of action in SLE treatment.

## 3 DISCUSSION

In recent years, the role of cell death in disrupting immune tolerance and regulating immune responses has garnered considerable attention. There has also been burgeoning interest in examining the role of innate immune cells in the development of SLE. Previous research has indicated that glutathione peroxidase 4 mediated ferroptosis and mitochondrial RNA (mtDNA) stimulated pyroptosis in the neutrophils of SLE patients.^8^, ^24^ It is known that macrophages in lupus are characterized by defects in phagocytosing apoptotic cells, aberrant activation, and imbalanced polarization. However, the conditions of programmed death in macrophages in SLE remain poorly understood. Earlier studies reported elevated GSDMD mRNA levels in PBMCs of SLE patients, along with enhanced expression of inflammasomes and GSDMD in macrophages and podocytes in the kidneys of lupus mice, and application of the GSDMD inhibitor disulfiram proved effective in treating lupus‐prone mice.^15^, ^16^, ^24^ Nonetheless, the occurrence of pyroptosis in lupus macrophages, the upstream signaling pathways, and the interaction between macrophage pyroptosis and adaptive immune responses have not been previously explored. This study focused on the presence of elevated pyroptosis in macrophages/monocytes in the context of SLE. Our findings validated that LPS leakage from a compromised gut barrier induced GSDMD‐mediated pyroptosis via the TLR4/Caspase 11 pathways in MRL/lpr mice. We discovered that pyroptotic macrophages promoted the differentiation of naive B cells into plasmablasts and plasma cells, potentially exacerbating the pathogenesis of lupus. Furthermore, inhibiting Caspase 11 and repairing the gut barrier with antibiotics effectively suppressed the Caspase 11/GSDMD pathways, thereby alleviating the manifestations of lupus in mice.

The involvement of GSDMD in the pathogenesis of SLE remains contentious. Serving as a crucial mediator of an inflammatory form of cell death known as pyroptosis, GSDMD facilitates the release of various inflammatory cytokines and nuclear components from ruptured cells, initially suggesting its role as a promoter in SLE development.^25^ This hypothesis was supported by the observed elevated expression of GSDMD in individuals with SLE and further corroborated by the beneficial effects of GSDMD inhibitors in alleviating symptoms in lupus‐prone mice.^15^ However, research by Wang et al.^14^ highlighted a potential protective role of GSDMD in imiquimod (a TLR7 agonist)‐induced lupus model. Surprisingly, *GSDMD* knockout mice developed more severe lupus symptoms, including higher spleen/body weight ratios, increased proteinuria, and extensive renal and lung damage, alongside elevated mortality rates compared with imiquimod‐treated wild‐type mice.^14^ In contrast, *GSDMD* knockout ameliorated severe symptoms such as splenomegaly, proliferative glomerulonephritis, and interferon‐alpha signaling in the pristane‐induced lupus (PIL) murine model.^24^ Both murine models demonstrate that global knockout of GSDMD significantly impacts myeloid innate immune cells, including monocytes, macrophages, and neutrophils. However, these models exhibit divergent outcomes in myeloid cell responses: there is an expansion of myeloid cells in the imiquimod‐induced GSDMD knockout mice, whereas in the PIL model, there is a general reduction in these cells. This divergence highlights the complex and context‐dependent roles of GSDMD in SLE pathogenesis, suggesting that its function may vary significantly depending on the specific immune challenges and environmental factors present in different lupus models.

In canonical pyroptosis, inflammasomes such as NLRP3, NLRC4, AIM2, and Pyrin are activated and cleave pro‐Caspase 1 into active Caspase 1, which subsequently cleaves GSDMD proteins to form an active N‐terminus that perforates the cell membrane. In contrast, noncanonical pyroptosis involves direct binding of LPS to Caspase 4/5/11, triggering their cleavage independent of inflammasomes, leading to GSDMD activation and cell membrane perforation. Notably, the N‐terminus of GSDMD produced by activated Caspase 4/5/11 in noncanonical pyroptosis not only mediates cell membrane lysis and pyroptosis but can also activate the inflammasome and Caspase 1, thereby potentially initiating a Caspase 1‐dependent canonical pathway.^10^ This study identified increased activation of both Caspase 1 and Caspase 5/11 in the PBMCs of SLE patients and in the kidneys of lupus mice, indicating the coexistence of both pyroptosis pathways in SLE. However, lupus serum and LPS were found to activate Caspase 11‐dependent but not Caspase 1‐dependent pyroptosis in macrophages. These findings suggest that noncanonical pyroptosis in SLE is triggered by serum LPS, while Caspase 1‐dependent pyroptosis may be induced by other stimuli. The elevated inflammasomes observed in the kidneys of lupus mice could explain the increased activation of Caspase 1, likely due to the intense inflammatory environment within renal tissue. Conversely, the PBMCs of SLE patients, which displayed normal levels of inflammasomes, suggest that the activation of Caspase 1 in these cells may result from noncanonical pyroptosis activation rather than direct inflammasome involvement. This indicates a complex interplay between canonical and noncanonical pathways in the pathophysiology of SLE, reflecting the diverse and multifaceted nature of immune responses in this autoimmune condition.

We discovered that pyroptotic macrophages significantly enhanced the activation of mature B cell subsets, which was not observed when LPS was administered alone or when macrophages were cultured without LPS. Additionally, WDL demonstrated an inhibitory effect on B cell differentiation in both in vivo and in vitro experiments, but it did not significantly impact T cell subsets. These findings suggest that Caspase 11 and GSDMD‐mediated pyroptosis primarily influence autoimmune responses through the regulation of B cell differentiation, independent of T cell involvement in SLE. This highlights a distinct pathway in which pyroptosis could modulate immune responses, particularly emphasizing its role in B cell dynamics within the context of autoimmune disease. In addition, WDL showed significant inhibitory effect on plasma cell differentiation in the absence of pyroptotic macrophages, indicating its ability of immune regulation. WDL is a natural metabolite of Eclipta and Wedelia. It inhibits LPS‐induced expression of Caspase 11 by inhibiting NF‐κB‐mediated transcription. WDL is an inhibitor of IKK, a key kinase that activates NF‐κB by mediating phosphorylation and degradation of IκBα.^26^ Previous studies showed that WDL has strong anti‐inflammatory and antioxidant abilities,^20^ while the regulatory ability to B cell differentiation has never been reported, which needs to be researched further in the future.

Patients with SLE exhibit significant differences in the composition, diversity, and abundance of gut microbiota compared with healthy individuals.^27^ This includes a consistent reduction in the Firmicutes to Bacteroidetes ratio across diverse ethnic populations.^28^, ^29^, ^30^, ^31^, ^32^ Recent clinical trials suggest that interventions aimed at restoring the gut microbiome to a healthier state can alleviate symptoms in SLE patients.^33^ Disrupted gut homeostasis may initiate inflammatory conditions and disrupt immune tolerance. With compromised epithelial integrity, bacteria or their products can migrate from the gut lumen into the peripheral circulation. This “leakiness” allows bacterial products such as LPS to circulate systemically, potentially enhancing immune cell stimulation. In our study, we observed compromised intestinal epithelium integrity and corresponding LPS translocation in lupus‐prone mice. To verify the assumption that the elevated serum LPS is due to the altered intestinal epithelium integrity, we treated lupus‐prone mice with combined antibiotics to eliminate intestinal flora. As expected, the serum LPS levels of the mice was decreased after treatment with antibiotics, accompanied by the epithelial tight junction proteins increased in intestines. This outcome implicates the gut microbiota as a key factor in maintaining intestinal integrity and modulating systemic inflammatory responses.

In terms of noncanonical pyroptosis mechanisms, extracellular LPS activates TLR4, upregulating the expression of precursors of the Caspase family and proinflammatory cytokines through the NF‐κB pathway. Intracellular LPS, meanwhile, activates Caspase 11, which specifically responds to cytosolic LPS and subsequently cleaves GSDMD, forming pores in the cell membrane and resulting in the secretion of various cytoplasmic molecules, such as IL‐1α.^25^ Previous research indicated that HMGB1 facilitates the translocation of LPS into the cytoplasm by binding to extracellular LPS and delivering it to the cytoplasm through RAGE‐mediated endocytosis and subsequent endo‐lysosomal membrane permeability.^22^, ^34^ However, in our study, LPS‐induced pyroptosis in macrophages occurred independently of HMGB1 mediation. Both LPS alone and combined LPS–HMGB1 treatments led to upregulation of Caspase 11 and GSDMD cleavages, as well as increased IL‐1α release. Furthermore, pretreatment with RAGE antagonists failed to prevent pyroptosis from the LPS–HMGB1 treatment. Conversely, the pyroptosis caused by LPS treatment and LPS–HMGB1 treatment was eliminated by the blockade of TLR 4 receptors, highlighting the critical role of TLR4 in mediating LPS‐driven pyroptotic responses.

Taken together, we have identified a novel pathway through which intestinal disorders may exacerbate the pathology of lupus. Elevated serum LPS, resulting from disruptions in the intestinal barrier, can induce Caspase 11/GSDMD‐mediated pyroptosis in the peripheral circulation and organs. This process creates an inflammatory environment and promotes the maturation and differentiation of B cells, thereby enhancing autoimmune responses in SLE. Our findings confirm that preventing LPS leakage and inhibiting Caspase 11 are effective strategies for alleviating lupus symptoms in mice. Given the controversial role of GSDMD in SLE pathogenesis, we propose Caspase 11‐targeted treatments as a potential therapeutic approach for SLE.

## 4 MATERIALS AND METHODS

### 4.1 Antibodies and reagents

A comprehensive list of the antibodies employed for immunofluorescence, western blot, flow cytometry, and mIHC can be found in Table S1. Drug information used for pyroptosis inducing or inhibition in vitro and lupus mice treatment are listed in Table S2.

### 4.2 Animals and experimental conditions

Female, 8 weeks C57BL/6 mice and MRL/lpr mice were procured from SLAC Laboratory Animal (Shanghai, China) and maintained in a specific pathogen‐free environment at the laboratory animal center of the Institute of Dermatology, Chinese Academy of Medical Sciences. Approval for the study was obtained from the Ethics Committee of the Institute of Dermatology, Chinese Academy of Medical Sciences (No. 2022‐DW‐017). All experiments were performed on age‐matched animals. 10 weeks old female MRL/lpr mice were divided into three groups in a random manner: the control group (*n* = 11), the WDL group (*n* = 12), and the antibiotics group (*n* = 12). The mice in the WDL group received a daily intraperitoneal dose of 10 mg/kg WDL for 9 weeks. Meanwhile, the mice in the control group were administered an equivalent vehicle daily. The mice in the antibiotics group were given aqueous solutions containing a combination of antibiotics (ampicillin 1 g/L, neomycin 1 g/L, metronidazole 1 g/L, and vancomycin 0.5 g/L) for 9 weeks. Biweekly, the mice were anesthetized using isoflurane to measure body weight and collect urine and blood samples. Subsequently, they were euthanized via rapid cervical dislocation to obtain kidney and spleen samples.

### 4.3 Clinical patient information

Serum and blood samples were obtained from six healthy individuals and nine patients diagnosed with SLE by 2017 EULAR/ACR criteria and consented to participate in our study. The clinical information is presented in Table S3. Informed consent was obtained from all participants. Kidney biopsies were obtained from an LN patient for diagnostic purposes. Renal tissue dissected adjacent to a renal tumor was obtained from a patient with a renal mass and was considered to be “normal.”

### 4.4 Isolation of human PBMCs and CD14^+^ cells

Blood samples from both individuals with SLE and healthy people were anticoagulated with heparin and subjected to density gradient centrifugation on Ficoll‐Paque (17144003; Cytiva, USA) for 30 min at 600 × *g* with the brake off. The mononuclear cells at the interface of two layers were carefully harvested and suspended in PBS. Centrifugation for 10 min at 500 × *g* and resuspending the sediment in lysing buffer (BL503B; Biosharp, China) to lyse remaining erythrocytes. After incubating the cells at room temperature for 5 min, an equal volume of PBS was added to halt the lysis process. After Centrifugation for 10 min at 500 × *g*, the sediment of mononuclear cells had been washed with PBS. Isolation of CD14^+^ monocytes from PBMCs was performed utilizing MojoSort™ Human CD14^+^ Monocytes Isolation Kit (BioLegend, USA) and were examined by TEM.

### 4.5 Isolation of mouse peritoneal macrophage

C57BL/6 mice were euthanized, and the abdominal area was sterilized by 70% ethanol. Subsequently, 10 mL of sterile PBS was carefully administered intraperitoneally. Following gentle abdominal massage, approximately 80% of the injected volume was successfully retrieved. Peritoneal macrophages were then isolated by adjusting the peritoneal suspensions to a concentration of 3 × 10^5^ cells/mL in RPMI 1460 and seeding them into 24‐well culture plates. The macrophages were cultured at 37°C for 2 h to adhere to the plastic surface. Upon completion of this incubation period, the supernatants containing nonadherent cells were gently aspirated after three times of PBS washing. The entire process was performed in a sterile environment.

### 4.6 Mouse CD4^+^ T cells and pan B cells isolation and activation

Spleens were extracted from C57BL/6 mice and CD4^+^ T cells and CD19^+^ B cells were isolated separately using magnetic cell sorting systems (MojoSort™ Mouse CD4 T Cell Isolation Kit and MojoSort™ Mouse Pan B Cell Isolation Kit; BioLegend). The isolated cells were cultured in RPMI 1640 culture medium with 10% FBS, 1% penicillin, 1% streptomycin, and 2‐mercaptoethanol at 37°C in a 5% CO_2_‐humidified incubator. CD4^+^ T cells (1.5 × 10^6^ cells) were seeded in a 24‐well plate and then stimulated with anti‐mouse CD28 antibody (2 μg/mL) and plate‐bound anti‐mouse CD3 antibody (5 μg/mL). For CD19^+^ B cells, R848 (100 ng/mL) and murine IL‐4 (30 ng/mL) were used to stimulate naïve B cells (1 × 10^6^ cells were seeded in a 24‐well plate). These cells were treated separately or combined as follows: cocultured with peritoneal macrophages isolated as processed above, LPS (1 μg/mL), HMGB 1 (400 ng/mL), and WDL (5 μg/mL).

### 4.7 Gut barrier permeability determination in vivo

Mice that had been fasted for 8 h were orally gavaged with 4 kDa fluorescent dextran (R‐FD‐001; Xinqiao Biotechnology, Hangzhou, China) at a dose of 300 mg/kg to assess intestinal permeability in vivo. After 2 h, serum was obtained from the blood of mice by centrifugation. Fluorescence intensity of FITC in serum was then analyzed using a microplate reader with excitation at 485 nm and emission at 535 nm.

### 4.8 Assessment of renal injury

After execution, the left kidneys of mice were fixed in 10% formalin for 24 h. Subsequently, the kidneys were embedded in paraffin and cut into 4 μm‐thick sections using a section cutter. Following this, the H&E staining of sections were performed following the manufacturer's guidelines. Renal injury was measured by the Austin score as previously described.^35^

### 4.9 Incubation of macrophages with serum and microscopy imaging

J774A.1 or RAW264.7 cells were cultivated by DMEM culture medium (with 10% FBS, 1% penicillin, 1% streptomycin) with 20% PBS or C57BL/6 mice serum or MRL/lpr mice serum at 37°C in a 5% CO_2_‐humidified incubator. To examine the morphology of pyroptotic macrophages, cells were seeded in 15‐mm culture dishes and treated as above. Static bright‐field images were captured using a Nikon Eclipse Ti2. The Time‐lapse microscopy assay was recorded by DeltaVision Elite Cell Imaging System (GE Healthcare Life Sciences, USA).

### 4.10 Immunofluorescence

Murine kidney and small intestine sections from frozen OCT embedded tissues (5 μm) were subjected to three washes with PBS and then blocked for 30 min using Blocking Buffer (P0260; Beyotime, China). Subsequently, the sections were exposed to specified primary antibodies overnight at 4°C. Following this, they underwent a gentle PBS wash three times before being treated with secondary antibodies at 37°C for 1 h. Finally, the sections were stained with DAPI staining solution (C1005; Beyotime) at room temperature for 15 min. Images were captured with Olympus BX53 microscopy and data were analyzed with cellSense software (Olympus).

### 4.11 Multiplex immunohistochemistry

mIHC staining was conducted following the guidelines provided by the manufacturer (PerkinElmer; Opal® Kit). The procedure involved the sequential application of the following antibodies and fluorescent dyes: Caspase 4/Opal620, Caspase 5/Opal520, GSDMD/Opal590. Sections of the paraffinized tissue were cut at 4 μm thickness and heated at 60°C for 1 h, then rehydrated in xylene, 100% alcohol, 90% alcohol, 70% alcohol successively. Epitope retrieval solution was preheated for antigen unmasking, followed by inactivation of endogenous peroxidase through a 15‐min incubation in blocking buffer (P0100A; Beyotime). Subsequently, the sections underwent preincubation with the kit's blocking buffer and were then exposed to specified primary antibodies at 37°C for 2 h. Then, the sections were treated with HRP‐conjugated secondary antibodies (ab205722; Abcam) at appropriate concentration for 30 min at 37°C. Then, the opal dyes were applied to bond the primary antibodies. Slides were imaged and scanned using Mantra software. The images were batch‐analyzed by Inform software (PerkinElmer).

### 4.12 Quantitative RT‐PCR

To conduct quantitative real‐time PCR (RT‐qPCR) analysis, total RNA was isolated using RNA‐easy Isolation Reagent (R701‐01; Vanzyme, China) and subsequently underwent cDNA synthesis utilizing the HiScript III RT SuperMix (R323‐01; Vanzyme). RT‐qPCR was conducted by utilizing SYBR Green Supermix (Q711‐02; Vanzyme). β‐Actin was used as the reference for normalizing the relative expression levels of mRNA. The sequences of qPCR primers for TLR 2 (5′‐GCTCCTGCGAACTCCTATC‐3′ and 5′‐GCCGGACTCATCGTACTCC‐3′), TLR 4 (5′‐CCTGACACCAGGAAGCTTGAA‐3′ and 5′‐TCTGATCCATGCATTGGTAGGT‐3′), Caspase 11 (5′‐GTGGTGAAAGAGGAGCTTACAGC‐3′ and 5′‐GCACCAGGAATGTGCTGTCTGA‐3′), GSDMD (5′‐CCATCGGCCTTTGAGAAA GTG‐3′ and 5′‐ACACATGAATAACGGGGTTTCC‐3′), IL‐1α (5′‐CGCTTGAGTCGGCAAAGAAA‐3′ and 5′‐GAGAGAGATGGTCAATGGCAGA‐3′), IL‐1β (5′‐TGGACCTTCCAGGATGAGGACA‐3′ and 5′‐GTTCATCTCGGAGCCTGTAGTG‐3′) and actin (5′‐GTGACGTTGACATCCGTAAAGA‐3′ and 5′‐GCCGGACTCATCGTACTCC‐3′).

### 4.13 Western blot

After lysis of cells and tissues in cell lysis buffer (P0013B; Beyotime) supplemented with protease and phosphatase inhibitor cocktail (P1045; Beyotime), the proteins were extracted by 12,000 × *g* centrifugation for 18 min. BCA protein assay kit was employed to determine the protein concentration in the supernatant. After denaturation of protein by heating, equal amounts of total protein were loaded and separated by SDS‐PAGE and then electrophoretically transferred onto a NC membrane. The membranes were blocked and incubated overnight with primary antibody at 4°C. After washing in TBST, the membranes were exposed to HRP‐conjugated antibodies for 1 h at room temperature. After washing in TBST, the bands were visualized by the Tanon 5200 system (TANON SCIENCE & TECHONLOGY, China).

### 4.14 Flow cytometry data acquisition and analysis

Cells were treated with Fc blocker diluted in PBS for 15 min. After that, the cells were incubated with specific flow cytometry antibodies along with Aqua (BioLegend) for 45 min at 4°C to stain membrane molecules. Then, the cells were treated with Cytofix/Cytoperm™ Fixation (BD Biosciences) for intracellular staining. Following incubation with antibodies for intracellular staining, the cells underwent two PBS washes and were subsequently analyzed using flow cytometry. Data acquisition was performed with Cytek Aurora (Cytek Biosciences) and analyzed utilizing FlowJo software (Tree Star, Inc.).

### 4.15 ELISAs

Serum was obtained from blood samples by centrifugation at 2000 × *g* for 5 min. ELISAs kits (Cusabio, Wuhan, China) were employed to detect the levels of anti‐dsDNA antibody, IL‐1β, IL‐1α, and HMGB1. The procedures conducted in accordance with the provided instructions.

### 4.16 Cytotoxicity assay

Following the specified treatments, the supernatant was gathered and the LDH assay was carried out utilizing the LDH Cytotoxicity Assay Kit (C0016; Beyotime) under the instruction of kit's guidelines. The cytotoxicity percentages was calculated based on the maximum LDH release observed in cells treated with 1% Triton X‐100.

### 4.17 Analysis of proteinuria

Mouse urine samples were tested by Urine protein content Assay Kit (D799855; Sangon Biotech, China) and Creatinine (Cr) Assay kit (C011‐2‐1, Nanjing Jiancheng Bioengineering Institute, China).

Proteinuria was measured by the urine protein/creatinine ratio (PCR).

### 4.18 Statistics analysis

GraphPad Prism 9.0 was utilized for the statistical analysis. The data are presented as mean ± SD (standard deviation), with specific statistical information provided in the figure legend. A two‐sample *t*‐test was conducted to assess variances between the two groups, with statistical significance defined as *p* < 0.05. The correlation analysis between the two samples was conducted using Pearson correlation analysis.

## AUTHOR CONTRIBUTIONS

Yue Xin and Qianjin Lu conceived the study. Yue Xin executed the experiments and drafted the original manuscript. Qianjin Lu, Changxing Gao, Lai Wang, and Qianmei Liu edited the manuscript. All authors have read and approved the final manuscript.

## CONFLICT OF INTEREST STATEMENT

The authors declare no conflict of interest.

## ETHICS STATEMENT

All animal and clinical experiments were approved by the Ethics Committee of the Institute of Dermatology, Chinese Academy of Medical Sciences. The approval number for the animal experiments is No. 2022‐DW‐017. The approval number for the clinical experiments is No. 2021‐KY‐054. Written informed consent was obtained from all participants.

## Supporting information

Supporting Information

Supporting Information

Supporting Information

Supporting Information

## Data Availability

The data that support the findings of this study are openly available in “Figureshare” at https://doi.org/10.6084/m9.Figureshare.23659743.
