# Supplementary material for: Lipopolysaccharide released from gut activates pyroptosis of macrophages via Caspase 11‐Gasdermin D pathway in systemic lupus erythematosus
Source: MedComm (2020). 2024 Jun 14;5(6):e610. doi: 10.1002/mco2.610 (PMC11176733; doi:10.1002/mco2.610)
Supplement: Supplementary file 4 — Supporting Information [file MCO2-5-e610-s004.docx]

SUPPLEMENTARY MATERIAL

Lipopolysaccharide Released from Gut Activates Pyroptosis of Macrophages via Caspase 11-Gasdermin D Pathway in Systemic Lupus Erythematosus

*Running Ttile: LPS Activates Pyroptosis of Macrphages in Lupus*

Yue Xin^1,2,3^, Changxing Gao^1,2,3^, Lai Wang^1,2,3^, Qianmei Liu^1,2,3^, and Qianjin Lu^1,2,3*^

^1^ Hospital for Skin Diseases, Institute of Dermatology, Chinese Academy of Medical Sciences and Peking Union Medical College, Nanjing, China

^2^ Key Laboratory of Basic and Translational Research on Immune-Mediated Skin Diseases, Chinese Academy of Medical Sciences, Nanjing, China

^3^ Jiangsu Key Laboratory of Molecular Biology for Skin Diseases and STIs, Chinese Academy of Medical Sciences, Nanjing, China

*Correspondence should be addressed to:

Qianjin Lu, 12 Jiangwangmiao Street, Xuanwu, Nanjing, Jiangsu, China 210042.

Email: [qianlu5860@pumcderm.cams.cn](mailto:qianlu5860@pumcderm.cams.cn)

Index

[Supplementary material 1. Antibodies 3](#_Toc494228673)

Supplementary material 2. Reagents [5](#_Toc494228778)

Supplementary material 3. Patient information 6

Supplementary material 4. Figure S1 7

**Supplementary material 1 Antibodies**

**Table S1.** Antibodies used for WB, FCM, IF and mIHC staining

| Antibodies | Application | Dilution | Product code |
| --- | --- | --- | --- |
| Anti-human GSDMD | WB | 1:1000 | 39754, CST |
| Anti-Caspase 4 | WB/mIHC | 1ug/ml, 2ug/ml | ab25898, Abcam |
| Anti-Caspase 5 | WB/mIHC | 1:1000, 1:100 | ab40887, Abcam |
| Anti- Caspase 5 p20 | WB | 1:1000 | sc-393346, Santa Cruz |
| Anti-mouse GSDMD | WB | 1:1000 | ab209845, Abcam |
| Anti-mouse Cleaved GSDMD | WB | 1:1000 | 10137, CST |
| Anti-DFNA5/GSDME | WB | 1:1000 | ab215191, Abcam |
| Anti-mouse Caspase 11 | WB | 1:1000 | ab180673, Abcam |
| Anti-human/mouse β-actin | WB | 1:1000 | 4970S, CST |
| Anti-mouse IL-α | WB | 1:1000 | ab300499, Abcam |
| Anti-mouse cleaved IL-β | WB | 1:1000 | 63124, CST |
| Anti-rabbit IgG, HRP-linked antibody | WB | 1:2000 | 7074, CST |
| anti-Mouse HRP, Secondary Antibody | WB | 1:2000 | 17690387, Proteintech |
| Anti-mouse Caspase 11 | IF | 1:200 | NB120-10454, Novusbio |
| Anti-mouse GSDMDC1 | IF | 1:800 | NBP2-33422, Novusbio |
| Anti-mouse F4/80 | IF | 5 ug/ml | ab6640, Abcam |
| Anti-mouse Synaptopodin | IF | 1:1000 | sc-515842, Santa Cruz |
| Anti-mouse Zonulin-1 | IF | 1:200 | 16891365, Proteintech |
| Donkey anti-rabbit IgG Secondary Antibody, AF488 | IF | 2ug/ml | A-21206, Invitrogen |
| Donkey anti-rabbit IgG Secondary Antibody, AF594 | IF | 2ug/ml | A-21207, Invitrogen |
| Goat anti-rat Rhodamine | IF | 1:200 | AS040, ABclonal |
| anti-mouse CD3-FITC | FCM | 1ul/tube | 100204, Biolegend |
| anti-mouse CD4-Percp-cy5.5 | FCM | 1ul/tube | 116012, Biolegend |
| anti-mouse CD25-AF700 | FCM | 1ul/tube | 102024, Biolegend |
| anti-mouse IFN-γ-APC | FCM | 1ul/tube | 505810, Biolegend |
| anti-mouse IL-17A-BV421 | FCM | 1ul/tube | 563354, BD |
| anti-mouse IL-4-PE-CY7 | FCM | 1ul/tube | 504118, Biolegend |
| anti-mouse Foxp3-PE | FCM | 1ul/tube | 563101, BD |
| anti-mouse CD8-APC-CY7 | FCM | 1ul/tube | 557654, BD |
| anti-mouse CD62L-PE-CY7 | FCM | 1ul/tube | 104418, Biolegend |
| anti-mouse CD44-BV421 | FCM | 1ul/tube | 103040, Biolegend |
| anti-mouse CD44-AF700 | FCM | 1ul/tube | 103026, Biolegend |
| anti-mouse CXCR5 | FCM | 1ul/tube | 551960, BD |
| APC Streptavidin | FCM | 1ul/tube | 405207, Biolegend |
| anti-mouse PD-1-PE | FCM | 1ul/tube | 135206, Biolegend |
| anti-mouse CD45R (B220)-PE-CY7 | FCM | 1ul/tube | 103222, Biolegend |
| anti-mouse CD19 APC-CY7 | FCM | 1ul/tube | 557655, BD |
| anti-mouse CD138-BV421 | FCM | 1ul/tube | 142508, Biolegend |
| anti-mouse GL-7-AF647 | FCM | 1ul/tube | 144606, Biolegend |
| anti-mouse Fas(CD95)-PE | FCM | 1ul/tube | 152608, Biolegend |
| anti-mouse IgD-PerCP-CY5.5 | FCM | 1ul/tube | 405710, Biolegend |

WB: Western Blot, FCM: Flow cytometry, IF: Immunofluorescence, mIHC: Multiplex immunohistochemistry

**Supplementary material 2 Reagents**

**Table S2.** Drugs information used for pyroptosis inducing or inhibition in vitro and lupus mice treatment

| Drugs | Product code |
| --- | --- |
| LPS | HY-D1056, MCE |
| HMGB1 | HY-P73104, MCE |
| TLR2 inhibitor | HY-100461, MCE |
| TLR4 inhibitor | HY-11109, MCE |
| RAGE inhibitor | HY-19370, MCE |
| Wedelolactone | HY-N0551, MCE |
| Ampicillin | HY-B0522, MCE |
| Neomycin | HY-B0470, MCE |
| Metronidazole | HY-B0318, MCE |
| Vancomycin | HY-17362, MCE |

LPS: lipopolysaccharides, HMGB1: high mobility group box 1 protein, TLR: Toll-like receptor

**Supplementary material 3 Patients information**

**Table S3.** Clinical characteristics of SLE patients enrolled.

| NO. | Gender | Age | SLEDAI-2K | Medication |
| --- | --- | --- | --- | --- |
| 1 | M | 24 | 18 | GCs, TAC |
| 2 | F | 24 | 7 | GCs, HCQ, AZA |
| 3 | M | 26 | 18 | GCs, TAC, MMF |
| 4 | F | 30 | 14 | GCs, HCQ |
| 5 | F | 23 | 18 | GCs, HCQ |
| 6 | F | 34 | 4 | GCs, HCQ, TAC, MMF |
| 7 | F | 30 | 10 | HCQ |
| 8 | F | 22 | 22 | GCs |
| 9 | F | 20 | 16 | GCs |

SLE: systemic lupus erythematosus; NO.: Number; F: female; M: male; SLEDAI-2K: Systemic Lupus Erythematosus Disease Activity Index-2000; GCs: Glucocorticoids; HCQ: Hydroxychloroquine; MMF: Mycophenolate mofetil; TAC: Tacrolimus, AZA: Azathioprine

**Supplementary material 4**

**
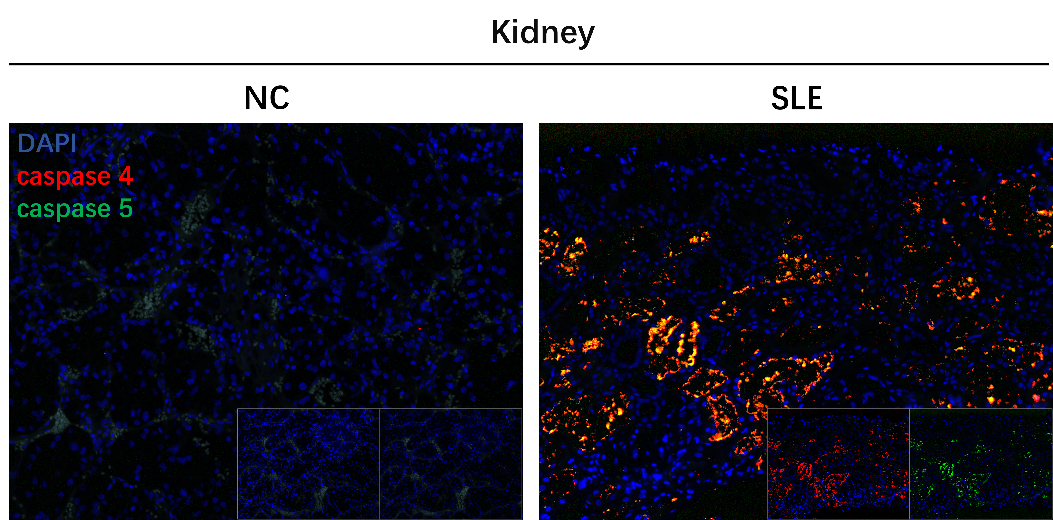
**

**Figure S1.** Multiplex immunohistochemistry of caspase 4 and caspase 5 in the renal biopsy samples from lupus nephritis patients and the renal peritumoral tissue from renal tumor patients as the control.
